# The Thorny Side of Addiction: Adaptive Plasticity and Dendritic Spines

**DOI:** 10.1100/tsw.2007.247

**Published:** 2007-11-02

**Authors:** Patrick J. Mulholland, L. Judson Chandler

**Affiliations:** Department of Neurosciences Center for Drug and Alcohol Programs, 67 President Street, Medical University of South Carolina, Charleston, SC 29425, USA

**Keywords:** alcohol, addiction, plasticity, NMDA receptors, dendritic spines, actin, postsynaptic density, PSD-95

## Abstract

Dendritic spines are morphologically specialized structures that receive the vast majority of central excitatory synaptic inputs. Studies have implicated changes in the size, shape, and number of dendritic spines in activity-dependent plasticity, and have further demonstrated that spine morphology is highly dependent on the dynamic organizational and scaffolding properties of its postsynaptic density (PSD). *In vitro* and* in vivo* models of experience-dependent plasticity have linked changes in the localization of glutamate receptors at the PSD with a molecular reorganization of the PSD and alterations in spine morphology. Chronic ethanol consumption results in adaptive changes in neuronal function that manifest as tolerance, physical dependence, and addiction. A potential mechanism supporting these adaptive changes that we recently identified is the homeostatic targeting of NR2B-containing NMDA receptors to the synapse. This increase is associated with and dependent on a corresponding increase in the localization of the scaffolding protein PSD-95 at the PSD, and with an actin-dependent increase in the size of dendritic spines. These observations led us to propose a molecular model for ethanol-induced plasticity at excitatory synapses in which increases in NR2B-containing NMDA receptors and PSD-95 at the PSD provide an expanded scaffolding platform for the recruitment and activation of signaling molecules that regulate spine actin dynamics, protein translation, and synaptic plasticity. This model is consistent with accumulating evidence that glutamatergic modulation of spine actin by the PSD plays a role in the aberrant plasticity of addiction.

*Be careful where you lie down boy*
*In this bed of roses.*
*Promises of petals then*
*You wake up on the other end.*

--The other end being the thorns.

These song lyrics[1], meant as a metaphor about the potential unpleasant outcome of an interpersonal relationship, could just as easily serve as a warning about the dangers of becoming involved with alcohol or drugs of abuse. For many individuals, while early drug experiences are often pleasurable, “this bed of roses” can quickly turn into a bed of thorns.

Dendritic spines were first described in the late 1800s by Spanish histologist Santiago Ramon *y* Cajal, who named them *espinas*, which in Spanish means thorns or spines[1]. Although initially discounted as artifacts of the Golgi staining procedure by his contemporaries, his idea (together with those of the Eugenio Tanzi) that these thorn-like structures could provide the cellular basis for learning and memory has become central to our concepts of how the brain changes and adapts in response to experience. This insight was remarkable, considering that virtually nothing was known at that time about the neuronal organization of the brain, and it would be decades before the discovery of synaptic communication. One of the more promising developments in addiction research in recent years has been the association of experience-dependent plasticity with the plasticity of addiction.

Synaptic plasticity is widely regarded as a substrate for many brain functions, including learning and memory[2]. An attractive idea that is gaining increasing support is that the persistence of drug and alcohol addiction requires changes in synaptic connectivity and efficacy that have their foundation in the plasticity of learning and memory formation. In agreement with this, evidence implicates experience-dependent changes in glutamatergic neurotransmission in the addicted brain[3]. These neuroadaptive changes may reflect manifestations of aberrant or inappropriate brain plasticity that are consistent with a glutamatergic basis of alcoholism and alcohol-related behaviors[4]. Alcohol addiction is characterized by craving for alcohol and compulsive alcohol-seeking behavior that often presents as a chronic relapsing disorder. Prolonged and excessive alcohol consumption is associated with the development of tolerance and physical dependence and a hyperexcitability withdrawal syndrome observed on abrupt cessation of consumption. The behavioral manifestations of chronic alcohol consumption likely reflect neuroadaptive changes that have a cellular, molecular, and structural basis, and one of the most important and challenging issues in addiction research is to understand how the brain modifies its structure and function in response to drug exposure. In this mini-review, we discuss a glutamatergic-based molecular model of chronic alcohol-induced plasticity in which chronic ethanol exposure leads to a homeostatic increase in synaptic NR2B-containing NMDA receptors. We propose that this selective targeting creates an expanded scaffolding platform for enhanced association and activation of signaling molecules at the postsynaptic density (PSD) that modulates actin-based spine morphology and contributes to the development and maintenance of addiction to alcohol and other drugs of abuse.

## NMDA RECEPTORS PLAY A CRITICAL ROLE IN SYNAPTIC PLASTICITY

The NMDA subtype of glutamate receptors is among the most widely distributed and abundant receptor-operated ion channels in the brain. NMDA receptors are heteromeric receptors composed of an obligatory NR1 subunit coassembled with a least one type of regulatory NR2A-D subunit[5]. The majority of NMDA receptors in the adult brain are composed of NR2A and NR2B subunits (with NR2C subunits primarily found in the cerebellum and NR2D subunits transiently expressed early in development). NMDA receptors mediate the slow component of excitatory postsynaptic potentials and are critical regulators of experience-dependent plasticity. Long-term potentiation (LTP) is a widely studied form of synaptic plasticity that is considered to be an *in vitro* correlate of learning and memory, and many forms of LTP are critically dependent on NMDA receptor activity[6]. NMDA receptors are also key regulators of neuronal survival and neuropathology, and have been implicated in disease states, such as schizophrenia, epilepsy, and addiction.

A number of recent studies indicate that NR2A- and NR2B-containing NMDA receptors play distinct roles in synaptic plasticity, with NR2B subunits being particularly associated with enhanced plasticity.

[1] *From the song ‘Mud’ by Richard Buckner.*

For example, NR2B subunit receptors mediate the so-called "critical period" of enhanced plasticity in the juvenile cortex and the adult brain [7,8,9], while overexpression of NR2B subunits lead to increased LTP *in vitro* and enhanced learning and memory *in vivo*[10,11]. This is consistent with differential regulatory processes that control the function and membrane expression of NR2A- vs. NR2B-containing NMDA receptors[12]. Thus, observation of enhanced expression of NR2B receptors at the synapse following chronic ethanol exposure is predicted to have important effects on synaptic plasticity[13,14].

## ETHANOL ALTERS THE MEMBRANE TRAFFICKING OF NMDA RECEPTORS

NMDA receptors have been implicated in ethanol-associated phenotypes, including sensitivity to ethanol intoxication, tolerance and physical dependence, withdrawal hyperexcitability, and craving and relapse to alcohol drinking[15,16]. Thus, NMDA receptor-dependent synaptic plasticity appears to underlie many aspects of ethanol-induced changes in brain function. Studies using a variety of preparations have clearly demonstrated that pharmacologically relevant concentrations of ethanol inhibit NMDA receptors[17]. This appears to occur through several distinct mechanisms. Ethanol has direct inhibitory effects on NMDA receptor activity (apparently by occupying a hydrophobic pocket(s) resulting in alterations in its gating properties)[18,19] and can alter channel function through changes in phosphorylation of the receptor subunits[16]. Such ethanol-induced changes in phosphorylation influence both NMDA receptor channel gating properties and their membrane trafficking. Together, the inhibitory effects of ethanol on NMDA receptor function contribute to the intoxicating actions of ethanol and, likely, to subsequent homeostatic adaptations.

While initial reports suggested that NMDA receptors in the membrane were relatively stable and required extended periods of time to undergo increases or decreases in number at the postsynaptic membrane, it was subsequently shown that they are indeed subject to dynamic membrane targeting and trafficking[20]. NMDA receptors exist in two spatially distinct pools within the dendritic membrane. One pool of receptors is localized at the postsynaptic membrane and the other is localized at nonsynaptic sites, which are referred to as synaptic and extrasynaptic receptors, respectively. Interestingly, synaptic and extrasynaptic NMDA receptors are often observed to couple to divergent signaling cascades with distinct and sometimes opposing actions, and their trafficking may also be differentially regulated. It should also be noted that extrasynaptic NMDA receptors appear to outnumber synaptic NMDA receptors by about 2 to 1 (based on total NMDA current flow measured before and after selective block of synaptic receptors)[13,21]. The C1 cassette located in the C-terminal tail of NR1 subunit splice variants influences the forward trafficking of NMDA receptors through the ER, and changes in expression of NR1 splice variants lead to changes in their expression and insertion into the membrane[20,22]. Although it is possible that newly synthesized receptors are inserted directly into the postsynaptic membrane, this is considered unlikely as receptor endocytosis-exocytosis appears to occur at specialized zones that are located extrasynaptically[23]. It also appears that the lateral movement of NMDA receptors through the membrane is an important mechanism for the delivery of NMDA receptors to the synapse[24]. While NMDA receptors are localized in clusters at the synapse through protein-protein interactions with components of the PSD, the mechanism for anchoring and clustering NMDA receptors at extrasynaptic sites is unclear, but may involve association with lipid raft domains.

Previous studies in a variety of preparations have shown that NMDA receptor number and/or function is increased following prolonged exposure to ethanol[15,25]. This increase presumably occurs as a homeostatic adaptive response to the prolonged suppression of NMDA receptor activity by ethanol and appears to be selective for NMDA receptors, as AMPA receptors and/or currents do not change. It was recently demonstrated that chronic exposure of cultured hippocampal neurons to ethanol increases NR2B-containing NMDA receptors selectively at the synapse without altering the levels of extrasynaptic NMDA receptors[26]. This is in contrast to the acute effects of ethanol that have been reported to induce a selective endocytosis of NR2A, but not NR2B-containing, NMDA receptors[27].

## DENDRITIC SPINES ORGANIZE SIGNALING NETWORKS AND ARE SITES OF STRUCTURAL PLASTICITY

Excitatory neurotransmission in the central nervous system takes place mainly at dendritic spines[28]. These structures are considered to be the primary location of synaptic plasticity in the brain. They possess the electron-dense PSD, which is a fibrous substructure that contains clusters of neurotransmitter receptors, scaffolding proteins, and a surprisingly large and diverse array of signaling molecules. Research has shown that the organization of the PSD is highly dynamic and plays an important role in regulating NMDA-dependent synaptic plasticity. NMDA receptors embedded within the PSD form macromolecular complexes with intracellular proteins through protein-protein interactions[29]. The cytoplasmic C-terminal tail of NR2 subunits bind to PSD-95/disc large/ZO-1 (PDZ) domains of a family of related PSD proteins known as membrane-associated guanylate kinases (MAGUKs). The prototype of this family of proteins is PSD-95. PSD-95 immobilizes NMDA receptors at the PSD and plays a critical organizational role through interaction with other proteins via multiple binding domains[30]. PSD-95 binds with scaffolding and cytoskeleton proteins, adhesion and signaling molecules, and glutamate receptors[30]. Importantly, up-regulation of the expression of PSD-95 occurs during the late phase of LTP when new and/or enlarged spines accumulate stable populations of glutamate receptors[31,32]. PSD-95 regulates AMPA and NMDA receptor trafficking, especially during experience-dependent plasticity and LTP[33,34]. Thus, PSD-95 complexes represent a network of molecular components providing compartmentalization and specificity for both temporal and spatial organization of signaling pathways.

Once observed in fixed tissue as static and immobile structures, advances in live-cell imaging have shown that dendritic spines are highly motile structures that can rapidly change size and shape, and it is now clear that spine structure is linked to LTP[35]. For example, long-lasting changes in synaptic efficacy during LTP is correlated with altered spine size, the emergence of new spines, and the perforation of the PSD (thought to reflect spine head splitting and eventual synapse duplication)[36,37,38]. In addition to serving as points of synaptic connectivity, two functions of mature dendritic spines are to regulate the flow of calcium from the spine into the dendritic shaft during transient increases in free calcium, and to organize and compartmentalize the PSD cytoskeletal-associated signaling complex[15,39,40]. Both of these functions are significantly influenced by the shape and size of the spine. Therefore, activity-dependent modifications in spine morphology are thought to contribute significantly to metaplasticity (this term was coined by Abraham and Bear[41] to describe changes in the ease with which subsequent plasticity can take place, and is often referred to as the “plasticity of plasticity”). An intriguing idea that is consistent with observations that chronic ethanol exposure increases spine size is that while ethanol can initially co-opt the processes of synaptic plasticity, with time and repeated ethanol exposure-withdrawal cycles, the ethanol-induced structural changes become entrenched and increasingly resistant to reversal (e.g., impaired metaplasticity) (see Fig. 1).

Behavioral experiences can alter the function of individual synapses or entire networks through changes in spine morphology, and activity-dependent changes in spine density and shape are correlated with behavioral alterations[42]. Exposure of rats to an enriched environment or training on a spatial learning task leads to increases in spine density[43]. Similarly, chronic stress[44,45], as well as changes in sex hormones[46,47], have been shown to alter spine size and number, while spine dysmorphology has been implicated in many mental retardation disorders, including Fragile X Syndrome[48,49]. Long-lasting changes in spine density and morphology have also been shown to occur in rats following repeated amphetamine exposure[50,51]. The appearance of these “structural modifications” within the addiction circuit temporally parallels the development of sensitization to the locomotor activating effect of drugs. Postmortem analyses of brains from alcoholics and from animals chronically exposed to ethanol have, for the most part, reported selective neuronal loss and a simplification and reduction in synaptic complexity[15]. Changes in spine morphology have also been noted in animal studies. However, while these studies are informative in relation to ethanol-related pathology, their interpretation is confounded by extraneous factors, such as very long-lasting ethanol exposure periods, nutritional insufficiency, and neuronal cell death. Surprisingly, to our knowledge there are no comprehensive *in vivo* studies of spine

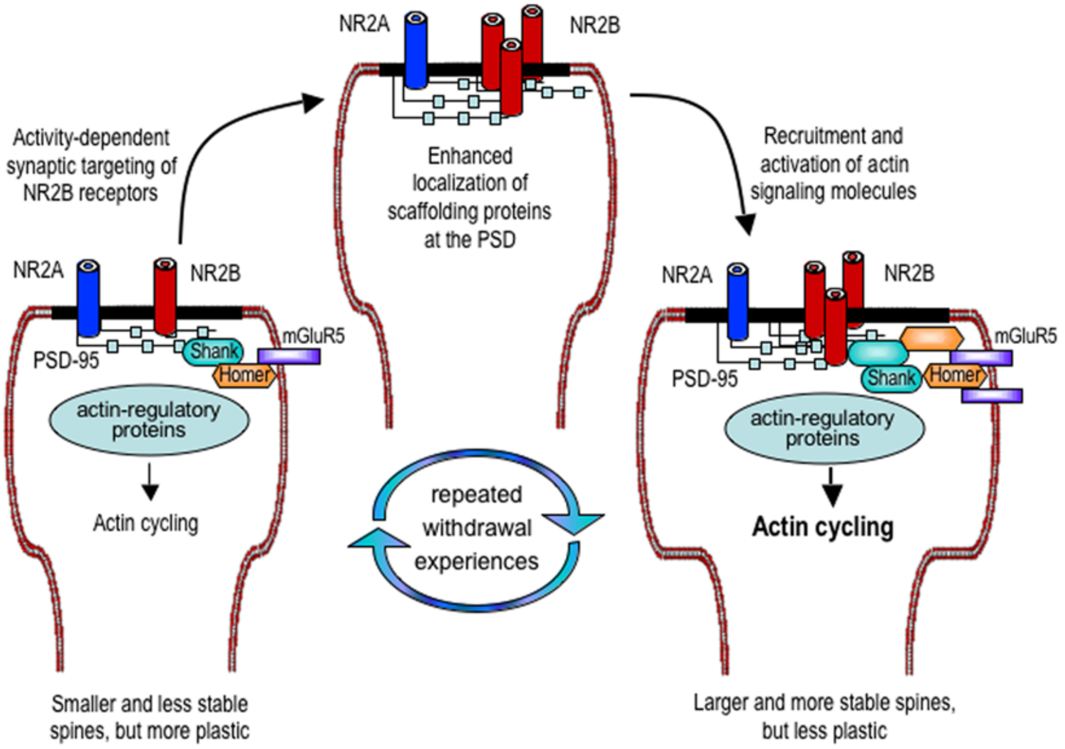

**FIGURE 1**. Model of ethanol-induced plasticity at glutamatergic synapses. In this model, increased targeting of NMDA receptors and PSD-95 to the PSD is the primary event in what is conceptually viewed as a linear multistep process of ethanol-induced plasticity. Tonic inhibition of NMDA receptor activity by ethanol leads to a compensatory increase in the synaptic expression of NR2B-containing NMDA receptors[26,103]. This increase is accompanied by, and dependent on, a parallel increase in the synaptic expression of PSD-95[14,103]. The multifunctional scaffolding properties of PSD-95 and NR2B-containing NMDA receptors provide an expanded scaffolding platform for further recruitment of components of the PSD signaling complex. Thus, the organizational properties of the PSD and its ability to undergo dynamic reorganization are central features of ethanol-induced changes in synaptic efficacy. Expansion of the scaffolding capacity of the PSD leads to increased recruitment and/or activation of actin regulatory proteins that may promote increases in spine size. An additional consequence of enhanced expression of NR2B-containing NMDA receptors and PSD-95 at the synapse may be changes in local protein synthesis and synapse-to-nuclear signaling that is coupled to translational-regulatory proteins of the PSD complex. Ethanol-induced increases in spine size may be an important component of synaptic remodeling associated with alcohol dependence and tolerance. Furthermore, successive cycles of alcohol exposure and withdrawal, which in animal models has been shown to promote alcohol-related behavioral plasticity, may act to further drive the formation of abnormally large spines. These aberrant changes in spine morphology may contribute to the development of alcohol tolerance and dependence, as well as contributing to the "hard wiring" of the addictive behavior that is akin to learning and memory type processes.

morphology that have examined short chronic alcohol exposure paradigms that avoid the confounds of pathology and/or nutritional insufficiency.

## THE PSD SIGNALING COMPLEX REGULATES ACTIN-DEPENDENT SPINE MORPHOLOGY

In recent years, it has become clear that the morphology of dendritic spines plays an important role in controlling the function of individual synapses. Dendritic spines vary by shape and size (i.e., mushroom, thin, and stubby), with the mushroom shape representing mature, stable spines that contribute to strong synaptic connections[52]. In contrast, smaller, less stable spines (e.g., stubby) contribute to weak synaptic connections, but are more plastic (Fig. 1). The relationship between morphology and function has led to the suggestion that mature, mushroom spines are "memory" spines, whereas immature, smaller spines are

"learning" spines[52]. In addition to the morphology of the spine head, recent evidence suggests that spine neck geometry may influence plasticity[53]. It is thought that the thinner neck in immature spines may reduce calcium leak into the dendrite, leading to a greater chance of LTP. Indeed, smaller spines show greater increases in calcium and preferential induction in LTP when compared with their more mature counterparts[54,55]. Morphological plasticity of dendritic spines depends on an intact spine cytoskeleton that is characterized by a dense meshwork of actin-containing microfilaments[56,57]. Spines also contain high levels of cytoskeletal-associated proteins that cross-link actin filaments to the plasma membrane and to the PSD itself. Of special note is that the vast majority of the scaffolding and signaling molecules associated with the PSD are involved in regulating actin cycling and dynamics of the spine[53,58]

Actin-dependent reorganization of spine size and shape has been strongly linked to the plasticity of learning and memory, and more recently to addiction. Studies using cell culture models and mice with gene deletions have clearly demonstrated that the scaffolding and organizational activity of the PSD is critical in regulation of actin cycling, spine morphology, and associated plasticity. Whether modulation of spine actin cycling plays a role in psychostimulant-induced changes in spine morphology as reported by Robinson and Kolb[59] is not known. However, it was recently shown that withdrawal from repeated cocaine administration induces a restructuring of actin-actin binding protein complexes, which in turn increases actin cycling[60]. Furthermore, this increase in actin cycling appeared to modulate cocaine-induced reinstatement. While similar studies on the effects of chronic alcohol on spine actin cycling have not yet been carried out, recent studies in flies and mice have revealed that alterations in regulatory components of actin cycling critically impact ethanol-related behaviors and plasticity[61,62]. In addition, changes in alcohol-responsive genes associated with plasticity and actin cycling were demonstrated in the mesocorticolimbic system, specifically within the nucleus accumbens and ventral tegmental area[63,64]. Chronic ethanol was also recently reported to increase the size of dendritic spines in a subpopulation of neurons in the nucleus accumbens of alcohol-preferring P rats[65]. It is also noteworthy that a recent study examining the genetic basis for alcohol preference in mice reported an over-representation of proteins involved in regulation of the actin cytoskeleton in the alcohol-preferring lines[66]. These data point to changes in gene expression profiles that suggest neuroadaptation in the reward circuitry, lending evidence for alcohol-induced persistent changes in subcellular constituents controlling spine morphology and synaptic plasticity.

Cellular actin exists as either polymerized filamentous actin (F-actin), the major constituent of the spine cytoskeleton, or as free monomers known as globular actin (G-actin). The level of F-actin in the spine is the product of the local balance between the rate of actin polymerization and depolymerization (i.e., addition or removal of G-actin) (see Fig. 2). Modulation of actin cycling can occur through interactive processes that involve controlling the rate of actin polymerization at the barbed end, regulating the rate of actin depolymerization at the blunt end, and regulating actin nucleation (e.g., branching of F-actin filaments). This assembly and disassembly (e.g., actin cycling) is under the control of actin regulatory and binding proteins (e.g., ADF/cofilin, profilin) that are themselves regulated by the PSD signaling network. In addition to proteins that regulate rates of polymerization/depolymerization, some actin regulatory proteins stabilize F-actin by binding to actin filaments. In general, actin stabilizers promote increases in the growth of F-actin filaments by reducing the rate of depolymerization. F-actin is organized into bundles or networks depending, in part, on the concentrations of cross-linking proteins, such as α-actinin[67]. Bundles of F-actin have been reported in spine necks where α-actinin concentrations are elevated. In contrast, networks of F-actin are found in spine heads and have been linked to postsynaptic NMDA receptors[68]. Dynamic changes in the actin cytoskeleton in both the head and neck regulate the capacity of dendritic spines to undergo changes in morphology[69]. Thus, spines can undergo changes in their morphology through activity-dependent changes in actin cycling. However, it is important to note that increases or decreases in actin cycling do not necessarily translate into a change in the total amount of F-actin in the spine. For example, an increase in the rate of actin polymerization is often balanced by an increase in the rate of depolymerization, with the result that there is no net change in the total amount of F-actin (or in the ratio of F/G actin). In addition, an increase in the amount of F-actin

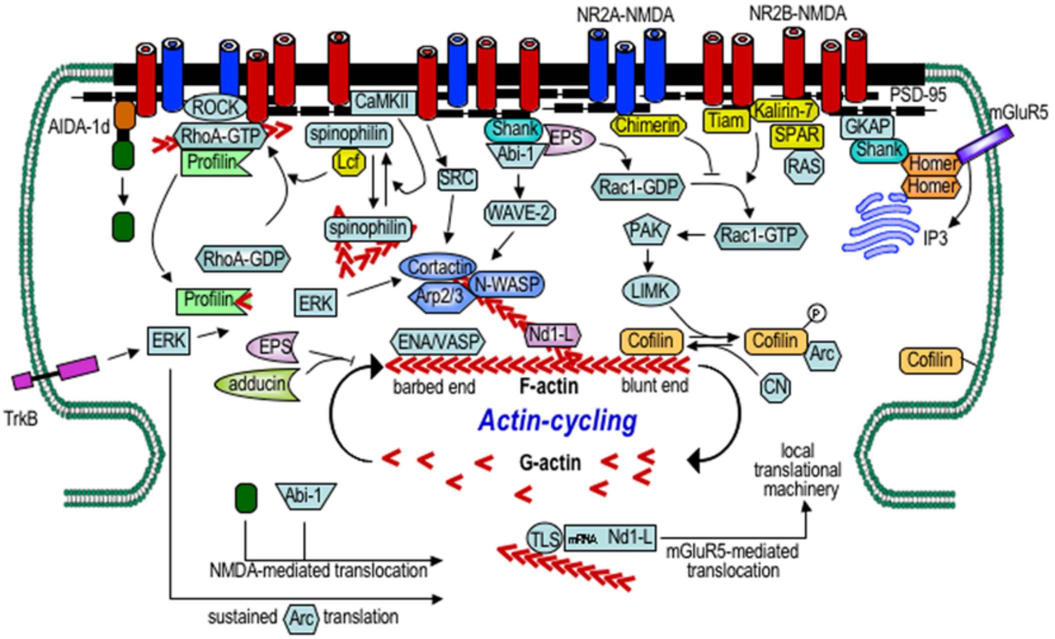

**FIGURE 2**. Schematic diagram of spine actin cycling that emphasizes the role of NMDA receptors and PSD-95 as scaffolding platforms for localization of actin regulatory proteins. One pathway of NMDA receptor control of actin depolymerization is the Rac-PAK-LIMK-cofilin signaling cascade. NR2B subunits specifically scaffold with Rac-GEFs (e.g., Tiam-1 and Kalirin-7) that activate Rac1, while NR2A receptors selectively scaffold the Rac-GAP Chimerin that inhibits Rac1 activation. A second regulatory pathway for NMDA control of actin cycling involves the regulated association of ROCK-RhoA complex with NMDA receptors. CaMKII, which preferentially associates with NR2B subunits, promotes the dissociation of spinophilin from actin, leading to activation of RhoA (via the RhoA-GEF Lcf). A third pathway involves regulation of the cortactin-Arp2/3-N-Wasp nucleation complex. NMDA receptors can activate SRC that in turn inhibits nucleation by phosphorylating cortactin on a specific tyrosine residue. In contrast, ERK stimulates nucleation by phosphorylating cortactin on a serine residue. Abi-1, which forms a scaffolding complex with NMDA receptors, Eps and Shank, stimulates nucleation via the WAVE-2 complex. Eps8 can modulate actin cycling by acting as an end-capping protein and by enhancing Rac1 activity. Also depicted are translational regulatory proteins associated with the PSD that regulate plasticity and spine morphology (see text).

may not reflect an increase in total actin if there is a corresponding reduction in the levels of G-actin. Finally, while changes in the amount of F-actin can have important effects on synaptic plasticity through alterations in spine morphology, changes in actin cycling, in and of itself, can also play an important role in plasticity since it is the dynamic turnover of F-actin that allows the spine to undergo changes in shape that can occur independent of changes in size. Changes in actin cycling can also impact signaling processes and neurotransmitter receptor functions independent of effects on morphology. Thus, to fully understand the effects of ethanol on spine actin, it is important to determine not only levels of F- and G-actin, but also changes in proteins that regulate actin cycling.

As noted above, NMDA receptors modulate actin cycling and spine remodeling. The key pathways of NMDA receptor coupling to actin cycling are depicted in the schematic cartoon shown in Fig. 2. For an in-depth discussion of these pathways, the reviewer is referred to several recent reviews[58,70]. In addition to NMDA receptor activity, growth factors and other extracellular signals affect actin cycling and spine morphology through activation of the Rho family of small GTPases[71]. In particular, RhoA and Rac1 profoundly influence morphology by inhibiting or promoting the growth and stability of spines. For example, inhibition of Rac1 produced elongated spines and blocked growth of spine heads, whereas inhibition of Rho resulted in protrusive motility of new spines[72]. Rho activity regulates clustering of the actin binding proteins spinophilin and neurabin with F-actin in spines[73]. Moreover, NMDA receptor activation regulates Rho sequestering at excitatory synapses, thus controlling profilin and actin depolymerization[74]. Studies have previously demonstrated acute actions of ethanol on actin cycling in non-neuronal cells, and recent evidence suggests that ethanol may also affect actin dynamics in behaving animals and in cerebellar neurons. In the fly, expression of the small GTPases Rho and Rac produced behavioral tolerance to the sedating effects of acute ethanol intoxication[61]. Knockdown of Eps8 in mice, which is involved in both activation of Rac and actin barbed-end capping, resulted in increased voluntary ethanol consumption, aberrant NMDA receptor currents, and resistance to changes in F-actin in response to acute ethanol[62].

An underlying principle of our model of chronic ethanol-induced plasticity is that it requires a dynamic PSD that can undergo activity-dependent expansion. Therefore, disruption in key proteins that are required for the adaptability of PSD will impede ethanol-induced plasticity. Homer proteins (Homer1-3) are scaffolding proteins of signaling complexes at the PSD[75,76] that interact with inositol trisphosphate and ryanodine receptors, Group I metabotropic receptors (mGluR1 and mGluR5), and NMDA receptors[77,78]. In addition, Homer proteins are thought to cooperate with Shank to control the structural organization of the PSD and have been implicated in regulating activity-dependent changes in spine morphology (see Fig. 2)[53,79]. It has been reported that mice with genetic deletion of Homer2 do not develop tolerance to repeated exposure of ethanol[80]. Similarly, it was recently reported that *drosophila* homer mutant flies exhibit increased sensitivity to the sedative effects of ethanol and failed to develop normal rapid tolerance[81]. Thus, the failure of Homer2 knockout mice to develop tolerance to the sedative actions of ethanol may relate, at least in part, to an inability of the PSD to support increases in NMDA receptors and actin-based spine remodeling. In the absence of a Shank-Homer2 complex, there may be deficits in the ability of the PSD to support homeostatic increases in NMDA receptors, resulting in a lack of tolerance development. This would be consistent with observations that the Shank-Homer2 complex modulates NMDA receptor localization at the PSD and spine morphology. In addition, while there may be an increase in NMDA receptors at the PSD in the Homer2 knockout mice in response to repeated alcohol exposure, the absence of the Shank-Homer2 complex may disrupt the ability of the PSD to support actin-based remodeling. However, while Homer2 knockout mice were shown to have reduced basal levels of membrane-associated NMDA receptors compared to wild type[80], it is not known whether chronic ethanol-induced up-regulation of NMDA receptors is attenuated in the Homer2 knockout mice. Thus, while there remain many unanswered questions, studies utilizing mouse lines with genetic disruptions in scaffolding and signaling components of the PSD may provide important insight in the role of spine remodeling in addictive processes. Of particular interest will be future studies in mouse models of genetic mental retardation disorders that are characterized by alterations in spine actin cycling and spine morphology, and impaired long-term synaptic plasticity.

## THE PSD SIGNALING COMPLEX MODULATES PLASTICITY VIA CONTROL OF ACTIVITY-DEPENDENT LOCAL AND NUCLEAR PROTEIN SYNTHESIS

In addition to scaffolding and recruitment of proteins of the spine actin regulatory network, recent studies have identified proteins of the PSD that regulate plasticity through activity-dependent changes in protein translation. These proteins undergo synapse-to-nuclear translocation in response to activation of NMDA receptors. Thus, similar to the enhanced scaffolding and activation of actin regulatory proteins, ethanol-induced increases in NMDA receptors and expansion of the scaffolding capacity of the PSD may lead to increases in the association of translational regulatory proteins that in turn influence plasticity through enhanced synapse-to-nuclear translocation. One such translational regulatory protein is Abelson interacting protein-1 (Abi-1). Abi-1 forms a complex with NMDA receptors Shank and Eps8 at the PSD (see Fig. 2)[82]. In addition to functioning as a regulator of actin cycling in the spine, Abi-1 undergoes translocation from the PSD to the nucleus in response to activation of NMDA receptors where it associates with the Myc/Max family of transcription factors. Thus, Abi-1 may play a dual role in activity-dependent plasticity through local regulation of spine actin dynamics and control of activity-dependent protein synthesis. The formation of an Abi-1 and Eps8 complex with NMDA receptors is of particular interest, as it was recently reported that Eps8 knockout mice are resistant to the actin-remodeling properties of NMDA and ethanol[62,83]. Eps8 knockout mice were more resistant to the sedative effects of ethanol and exhibited increased ethanol consumption compared to wild-type mice. This suggests that Eps8 may be a key modulator of cellular and behavioral responses to ethanol. Thus, consistent with our proposed model, chronic ethanol exposure may increase the localization of both Eps8 and Abi-1 at the PSD that could in turn increase the synapse-to-nuclear translocation of Abi-1 in response to enhanced NMDA receptor activity during ethanol withdrawal hyperactivity.

Another translational regulatory protein of the PSD that is associated with NMDA receptors is AIDA-1d. AIDA-1d has been shown to bind to the first two PDZ domains of PSD-95 via its C-terminal three amino acids[84]. Activation of NMDA receptors induces the cleavage of the N-terminal half of AIDA-1d that then undergoes synapse-to-nuclear transport where it regulates mRNA splicing (see Fig. 2). This appears to involve its transport to and association with Cajal bodies that are themselves associated with nucleoli. Cajal bodies contain the highest concentration of small nuclear ribonuclearproteins (snRNPs) in the nucleus and play a vital role in pre-mRNA processing including splice site recognition[85,86]. After their transport into the nucleus, newly formed snRNP accumulate in the Cajal bodies. This accumulation is contingent on snRNP binding to coilin. It has been proposed that AIDA-1 facilitates the biogenesis of snRNPs by modulating the interaction between coilin and snRNPs[87]. The N-terminal fragment of AIDA-1d generated by NMDA receptor–induced proteolytic cleavage contains a nuclear localization sequence (NLS) that is required for its transport from the PSD to Cajal bodies[84]. Thus, chronic ethanol induced increases in PSD-95 at the synapse may enhance the scaffolding of AIDA-1 at the PSD. The increase in localization of NMDA receptors and AIDA-1 at the PSD could promote increased nuclear translocation of the N-terminal fragment to Cajal bodies and thus impact mRNA processing and expression of splice variants.

Activity-dependent control of dendritic mRNA localization and local regulated translation has also increasingly been implicated in spine morphology and plasticity[49,88,89,90,91]. Transforming in liposarcoma (TLS) is an mRNA binding protein that has recently been shown to play an important role in regulating activity-dependent changes in actin dynamics and in spine morphology[92]. TLS appears to specifically bind mRNAs encoding actin regulatory proteins and is involved in rapid nuclear-to-cytoplasmic shuttling by binding mRNA in the nucleus and exporting spliced mRNAs as a ribonuclearprotein complex to the cytoplasm. In addition to high levels of expression in the nucleus, TLS also shows high levels of dendritic expression. Activation of mGluR5 receptors has been shown to induce the translocation of TLS from the underlying dendritic shaft into the spine where it is colocalized with PSD-95[93]. This is of particular interest, as mGluR5 receptors are increasingly implicated in spine morphology and in ethanol-related plasticity[49,94]. An mRNA transcript that has been identified as a TLS binding partner is mRNA encoding the actin-stabilizing protein Nd1-L[92]. Stimulation of mGluR5 leads to TLS-mediated translocation of Nd1-L mRNA to the spine that is accompanied by increases in the levels of Nd1-L protein. This translocation is dependent on an active actin cytoskeleton (e.g., blocking actin cycling with cytochalasin prevents dendrite-to-spine translocation of the TLS-RNP complex and increases in Nd1-L protein). This supports the suggestion that during activity-dependent spine remodeling, activation of NMDA receptors stimulates actin cycling, which releases actin-bound TLS[92]. As illustrated in Fig. 2, unbound TLS is then free to undergo mGluR5-dependent transport to the local translational machinery of the spine. The resulting increase in local synthesis of actin regulatory proteins, such as Nd1-L, may in turn promote spine stabilization. Therefore, chronic ethanol-induced increases in postsynaptic NMDA receptors, in conjunction with mGluR5 signaling, may alter TLS translocation and transcription of actin-related proteins, including increases in the expression of the actin-stabilizing protein Nd1-L. Interestingly, polyribosomes redistribute from dendritic shafts into spines with enlarged synapes during LTP[95], and activation of mGluR5 receptors has been observed to rapidly increase the aggregation of polyribosomes[96] and to increase spine size[97]. Thus, changes in NMDA-mGluR5–mediated translocation of TLS as a means of regulating synthesis of actin regulatory proteins locally at the spine could be particularly important in ethanol-induced plasticity. In addition, chronic ethanol-induced increases in PSD-95 may support expansion of the Shank-Homer2-mGluR complex. The resulting increases in signaling through NMDA and mGluR receptors could work together to promote increases in spine size in response to chronic ethanol by promoting increased synthesis of actin regulatory proteins. This association with mGluR5 signaling may be particularly relevant in light of recent studies implicating mGluR 5 receptors in ethanol- and cocaine-related behavioral plasticity[98,99,100,101,102].

## SUMMARY

In this mini-review, we have outlined a model in which alterations in the scaffolding and organizational properties of the PSD play a critical role in the development and maintenance of addiction. This model is consistent with increasing evidence suggesting that addiction results from the co-opting of plasticity mechanisms normally involved in the ability of the brain to modify its structure and function in response to changes in its environment. If, as data from recent studies strongly suggest, structural changes in dendritic spines represent the engagement of physiological mechanisms associated with learning and memory, entrapment of an individual in the "thicket of thorns" that is addiction may literally have a biological basis in spine morphology. Understanding how to approach and properly tend this prickly problem (rose garden) will be an important step in the development of effective treatments of addiction.

## ACKNOWLEDGMENTS

The authors' work discussed in this review was supported by NIAAA AA010983 and a grant from the Alcoholic Beverage Medical Research Foundation. P.J.M. is supported by National Research Service Award AA016450.
